# A Qualitative Review of Clinical Decision‐Making Using [^99m^ Tc]Tc‐Mercaptoacetyltriglycine Renal Scintigraphy in Patients With Malignant Ureteral Obstruction

**DOI:** 10.1002/jmrs.70029

**Published:** 2025-10-12

**Authors:** Akira Ohtsu, Seiji Arai, Tirso Peña, Yusuke Otani, Mai Onose‐Kato, Yusuke Tsuji, Tatsuhiro Sawada, Yuji Fujizuka, Yoshitaka Sekine, Hidekazu Koike, Tetsuya Higuchi, Kazuhiro Suzuki

**Affiliations:** ^1^ Department of Urology Gunma University Graduate School of Medicine Maebashi Gunma Japan; ^2^ Department of Medicine and Cancer Center, Beth Israel Deaconess Medical Center Harvard Medical School Boston Massachusetts USA; ^3^ Department of Pathology Beth Israel Deaconess Medical Center Boston Massachusetts USA; ^4^ Harvard Medical School Boston Massachusetts USA; ^5^ Department of Diagnostic and Interventional Radiology Gunma University Graduate School of Medicine Maebashi Gunma Japan

**Keywords:** [^99m^Tc]Tc‐MAG_3_ scintigraphy, conservative treatment, hydronephrosis, malignant ureteral obstruction, radioisotopes

## Abstract

**Introduction:**

Urinary drainage (ureteral stenting or percutaneous nephrostomy) is commonly used for malignant ureteral obstruction (MUO), but optimal indications remain unclear. [^99m^Tc]Tc‐mercaptoacetyltriglycine (MAG_3_) renal scintigraphy assesses urinary tract obstruction and may help identify patients who can avoid drainage. The aim of this case series was to investigate the impact of urinary drainage guided by MAG_3_ findings on renal function in MUO patients.

**Methods:**

We retrospectively reviewed 44 MUO patients who underwent MAG_3_ scintigraphy between April 2020 and January 2022. Based on results, 29 patients underwent urinary drainage and 15 patients were treated conservatively. Patients were classified by MAG_3_ excretion pattern and followed by renal function, pyelonephritis and flank pain at 1, 2, 3 and 6 months.

**Results:**

Among the conservative group (*n* = 15), MAG_3_ patterns included non‐function (*n* = 7), delayed excretion (*n* = 7) and obstruction (*n* = 1). No patients developed renal deterioration or pyelonephritis, though one patient underwent drainage for contralateral flank pain. Among the drainage group (*n* = 29), MAG_3_ patterns included obstruction (*n* = 16), delayed excretion (*n* = 8), declined excretion (*n* = 3) and non‐function (*n* = 2).

**Conclusion:**

Fourteen of 15 patients treated conservatively after MAG_3_ scintigraphy experienced no renal complications during 6 months of follow‐up. MAG_3_ scintigraphy may support individualised decision‐making and help avoid unnecessary drainage. Conservative management may be appropriate for patients with a non‐functional MAG_3_ pattern.

## Graphical abstract

This case series evaluated the clinical utility of [^99m^Tc]Tc‐MAG_3_ renal scintigraphy in managing malignant ureteral obstruction (MUO). Renal scintigraphy‐guided decision‐making allowed safe conservative management in selected patients, particularly those with a non‐functional excretion pattern, and helped avoid unnecessary urinary drainage.

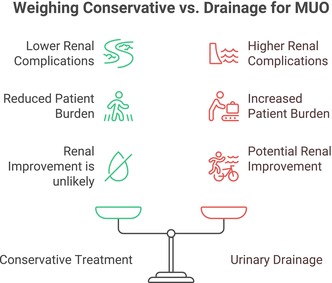

## 1 Introduction

Malignant ureteral obstruction (MUO) occurs in patients with advanced cancer and presents with hydronephrosis. MUO may increase the risk of renal dysfunction and pyelonephritis and cause low back pain. Although MUO is usually treated by urinary drainage (ureteral stent insertion or percutaneous nephrotomy), the exact indications for this treatment have not been determined [1]. In Japan, the reported median overall survival time in patients with MUO ranges from 210 to 226 days [2, 3]. Therefore, urologists need to preserve the quality of life for these patients as long as possible.

Urinary drainage increases the risk of complications while it alleviates urinary tract obstruction. For example, the insertion of a ureteral stent increases the risk of haematuria, painful voiding and frequent urination, and percutaneous nephrostomy might induce a further decrease in kidney function. Urinary drainage also increases the physical burden of surgery and anaesthesia. If unnecessary, urinary drainage has no benefit for patients with MUO; thus, urologists should avoid such procedures. The ‘no intervention’ option should also be discussed if the residual kidney function is poor [4]. Therefore, it is essential to evaluate the degree of ureteral obstruction and correctly assess residual renal function before performing surgical drainage.

Renal scintigraphy or renography using [^99m^Tc]Tc‐mercaptoacetyltriglycine (MAG_3_) is a relatively simple and non‐invasive test that only requires intravenous administration of MAG_3_ and can evaluate renal plasma volume and urinary tract obstruction. MAG_3_ scintigraphy is widely used to assess renal function, particularly when evaluating postoperative complications in renal transplant recipients [5]. In an animal model, MAG_3_ scintigraphy accurately identified potentially obstructed and non‐obstructed kidneys [6]. Paediatric urologists have also used MAG_3_ scintigraphy during the follow‐up period after pyeloplasty [7]. The aim of this case series is to investigate the impact of urinary drainage guided by MAG_3_ scintigraphy results on renal function in patients with MUO. In this case series, we performed MAG_3_ scintigraphy in patients with MUO to evaluate their residual renal function and the pattern of ureteral obstruction and evaluated the outcomes in patients deemed suitable for conservative treatment.

## 2 Patients and Methods

We retrospectively identified patients with MUO who underwent MAG_3_ scintigraphy between December 2019 and January 2022 at our institution, Gunma University. Urinary drainage was defined as the placement of ureteral stents or percutaneous nephrostomy tubes. Conservative therapy referred to observation without any urinary diversion. The MAG_3_ scintigraphy results were categorised into four patterns based on qualitative and semi‐quantitative assessment in accordance with international guidelines and literature [8]. First, non‐function pattern: defined as absent or markedly reduced radiotracer uptake in the affected kidney, with no appreciable cortical transit or excretion. Typically associated with a flat or low activity time–activity curve and split renal function < 10%. Second, obstruction pattern: defined by preserved radiotracer uptake and cortical transit but persistent retention in the pelvicalyceal system despite diuretic challenge. A drainage half‐time (T1/2) > 20 min was suggestive of obstruction. Third, delayed voiding pattern: defined as preserved function with slow yet progressive tracer excretion after furosemide administration, with delayed downslope on the renogram but no plateau. Fourth, functional decline pattern: characterised by reduced tracer uptake and delayed excretion compared to the contralateral side, indicating moderate impairment of function without meeting criteria for non‐function or definite obstruction. Interpreted in the clinical context, these classifications were based on visual inspection of the time–activity curve, tracer distribution and semi‐quantitative indices. Urologists recommended the treatment plan (urinary drainage or conservative treatment) based on the results of MAG_3_ scintigraphy: patients shared in the decision‐making process. In general, urinary drainage was recommended for patients with an obstruction pattern; not recommended for those with a non‐function pattern; based on clinical context, individualised decisions were made for cases with delayed excretion or reduced function.

Patients who received conservative treatment and were followed for up to 6 months were included in this case series. We extracted data from the medical records on age, sex, type of cancer and treatment, the serum creatinine level and estimated glomerular filtration rate (eGFR) at the time of MAG_3_ scintigraphy, hydronephrosis grade and status, renal volume, findings on contrast‐enhanced CT imaging and nephrography, excretion pattern seen on MAG_3_ scintigraphy, survival status and reasons for urinary drainage. We also examined changes in the serum creatinine level and eGFR over time, which had been used to decide whether to continue conservative treatment.

In all patients, 200 MBq of [^99m^Tc]Tc‐MAG_3_ (PD Radiopharma Inc., Tokyo, Japan) was administered. Dynamic planar images were acquired using a dual‐detector gamma camera (E‐CAM; Canon Medical Systems, Otawara, Tochigi, Japan) equipped with a low‐energy high‐resolution collimator and a [^99m^Tc]Tc 140 keV collection window for [^99m^Tc]Tc. A dynamic acquisition was performed for 30 min with a matrix size of 64 × 64 (180 frames at 10 s/frame), and 20 mg of furosemide was administered intravenously at 10 min after MAG_3_ injection [9].

We also explored the relationship between the pattern of obstruction seen on MAG_3_ scintigraphy and the outcomes in patients who underwent urinary drainage. In the conservative treatment group, we defined ‘success’ as no loss of renal function, pain or pyelonephritis during follow‐up and ‘failure’ as the development of such events. In the urinary drainage group, we defined ‘success’ as at least 10% improvement in eGFR before drainage and at least 1 month after drainage, and ‘failure’ as no improvement.

The relationship between renal contrast status on each side and treatment outcomes was evaluated in all patients who underwent contrast‐enhanced CT using the above‐mentioned definitions of ‘success’ and ‘failure’.

The study protocol was approved by the Gunma University Hospital Institutional Review Board (approval number 2114) and conducted in accordance with the ethical standards of the Declaration of Helsinki. The requirement for informed consent was waived by the same committee.

## 3 Results

**FIGURE 1:**
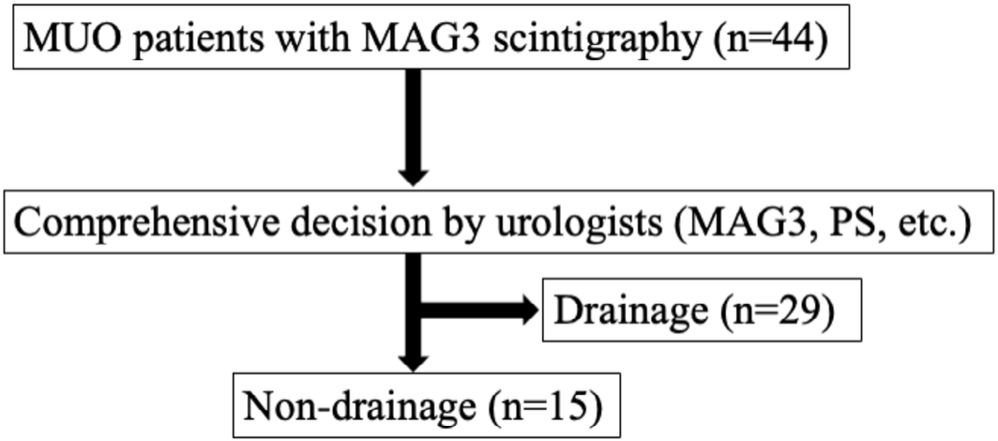
Flow chart showing the patient selection process. MAG_3_, [^99m^Tc]Tc‐mercaptoacetyltriglycine scintigraphy; MUO, malignant ureteral obstruction; PS, performance status.

Forty‐four consecutive patients with MUO underwent MAG_3_ scintigraphy during the quantitative review period. Twenty‐nine patients underwent urinary drainage and 15 were treated conservatively (Figure 1). In one case, drainage was not performed despite an obstruction pattern due to the poor performance status of the patient. Conversely, in two cases with a non‐function pattern, urinary drainage was performed at the strong request of the primary clinical department.

**FIGURE 2:**
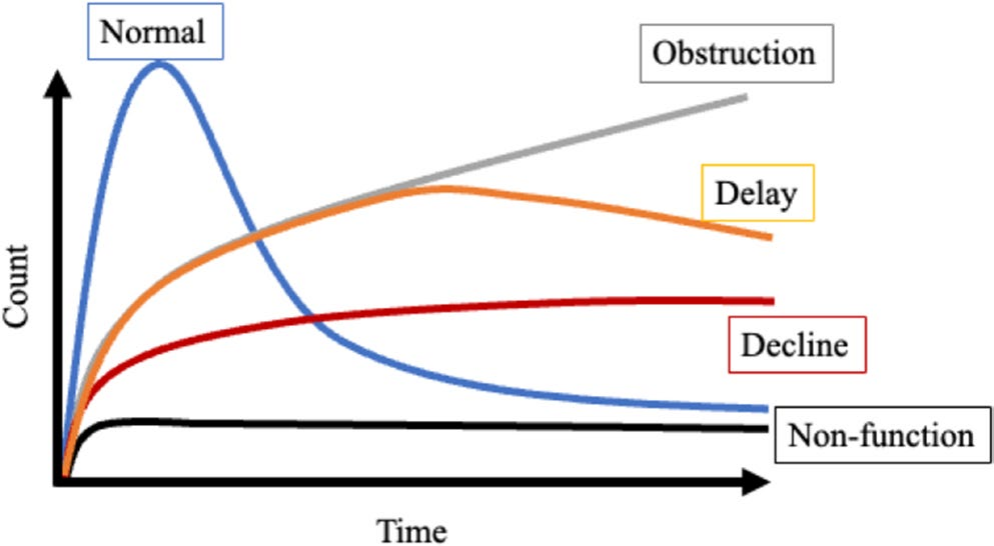
Schema showing the excretion patterns seen on [^99m^Tc]Tc‐mercaptoacetyltriglycine (MAG_3_) scintigraphy.

**FIGURE 3:**
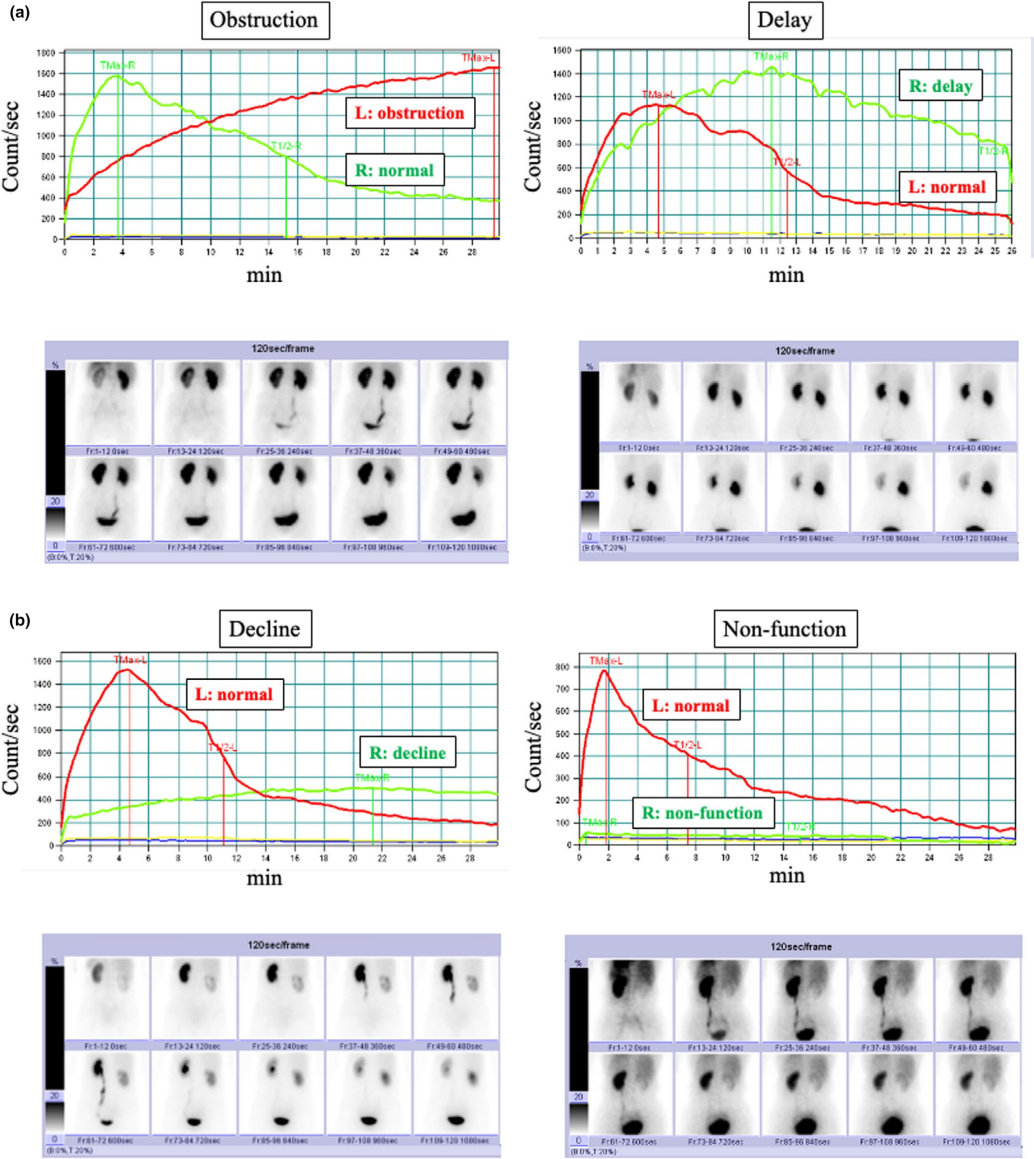
Representative excretion patterns from four types identified in this case series. (a) Obstructive pattern and delayed voiding pattern. (b) Declining excretion pattern and non‐function pattern. L indicates the left side (red), and R indicates the right side (light green).

**FIGURE 4:**
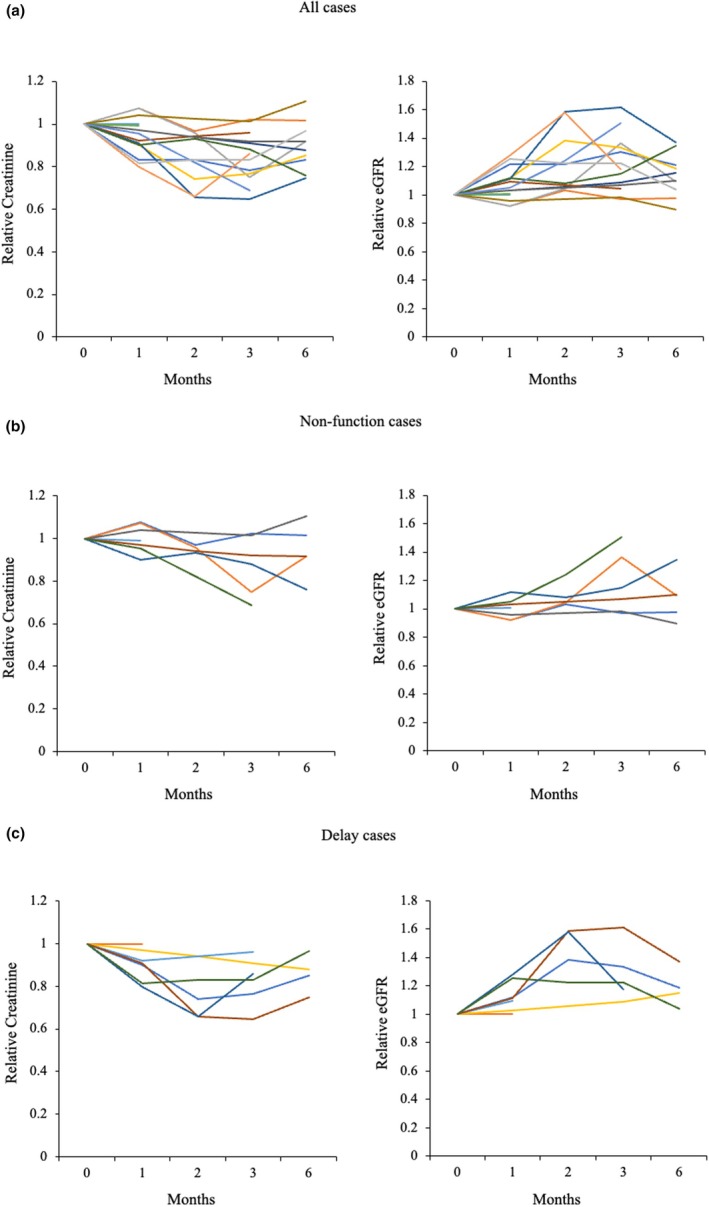
Changes in the relative serum creatinine level and estimated glomerular filtration rate. (a) All cases. (b) Cases with a non‐function pattern. (c) Cases with a delayed voiding pattern.

The 15 patients who received conservative treatment consisted of five men and 10 women with a median age of 72 years (Table 1). All 15 patients had hydronephrosis (left‐sided, *n* = 7; right‐sided, *n* = 7; bilateral, *n* = 1). The primary cancer sites included the uterus (*n* = 4), ovary (*n* = 2), stomach (*n* = 2), bladder (*n* = 2) and others (*n* = 5). Seven patients received chemotherapy and seven received best supportive care (BSC). The MAG_3_ excretion pattern showed no function in seven cases, delayed voiding in eight cases and obstruction in one (Figure 2). Representative patterns of the four types of excretion observed among these patients are shown (Figure 3). During follow‐up, three patients died of their primary cancer, and none developed pyelonephritis or renal failure. One patient underwent ureteral stenting for left‐sided hydronephrosis and low back pain that occurred during conservative treatment. In this patient, we assessed the outcome of right‐sided hydronephrosis that was treated conservatively. We analysed the serum creatinine level and eGFR at 1 month in two patients (2 subsequently died), at 3 months in three patients (1 subsequently died, 2 lost follow‐up) and at 6 months in 10 patients (Figure 4). No patient showed a significant decline in renal function.

**TABLE 1:** Patient demographic and clinical characteristics in a cohort managed with conservative treatment.

No.	Age (years)	Sex	Type of cancer	Therapy	Cr (mg/dL)	eGFR (mL/min/1.73 m^2^)	Hydronephrosis	Left kidney volume (cm^3^)	Right kidney volume (cm^3^)	Contrast‐enhanced CT	Laterality of CE	Grade of hydronephrosis (SFU)	MAG3 pattern	Death during follow‐up	Failure	Cause of failure
1	39	Female	Ovary	Chemo	0.6	88.3	Right	1687	2133	+	+	2	Obstruction	−	−	
2	73	Male	Osteosarcoma	Follow‐up	1.87	28.7	Left	851	1288	−	/	3	Non‐function	−	−	
3	73	Female	Uterine adenosarcoma	Chemo	0.96	43.9	Left	1013	991	+	+	3	Non‐function	−	−	
4	72	Female	Endometrial	Chemo	0.81	53.1	Left	932	986	+	−	2	Delay	−	−	
5	72	Female	Pancreas	Best supportive	0.36	128.5	Right	1020	1465	+	−	3	Delay	+	−	
6	74	Male	Stomach	Best supportive	1.19	46.8	Left	978	1000	+	+	2	Non‐function	+	−	
7	70	Female	Ovary	Follow‐up	0.74	58.9	Right	954	797	+	−	2	Delay	−	−	
8	72	Male	Bladder	Follow‐up	0.76	77.1	Bilateral	1621	1714	+	−	2	Delay	−	−	
9	80	Male	Lymphoma	Follow‐up	1.33	40.4	Right	1384	1557	+	+	2	Non‐function	−	−	
10	56	Female	Cervix	Radiation	0.75	61.9	Right	829	833	+	+	3	Non‐function	−	−	
11	63	Female	Stomach	Chemo	0.99	44.3	Left	647	649	+	+	2	Delay	−	−	
12	65	Male	Rectum	Chemo	1.33	43	Right	1121	1237	+	+	2	Non‐function	−	−	
13	35	Female	Bladder	Chemo	0.67	80.1	Left	926	1251	+	+	3	Non‐function	+	−	
14	63	Female	Cervix	Chemoradiation	0.79	55.3	Left	1749	1229	−	/	2	Delay	−	−	
15	73	Female	Lung	Chemo	0.59	74.5	Right	760	824	−	/	1	Delay	−	+	Left low back pain

Abbreviations: CE, contrast enhancement; CR, creatinine; CT, computed tomography; eGFR, estimated glomerular filtration rate; MAG_3_, [^99m^Tc]Tc‐mercaptoacetyltriglycine scintigraphy; SFU, Society of Foetal Urology grading system.

Based on this data, we summarised the relationship between the MAG_3_ excretion pattern and the treatment outcomes in all 44 patients (Table 2). Nine patients had a non‐function pattern, 17 had an obstruction pattern, 15 had a delayed voiding pattern and three had a functional decline pattern. We provided conservative treatment in seven of the nine patients with the non‐function pattern, one of the 17 with the obstruction pattern, seven of the 15 with the delayed voiding pattern and none of the three patients with the functional decline pattern. All other patients underwent urinary drainage. Conservative treatment was successful in seven of the nine cases with the non‐function pattern and unsuccessful in the remaining two cases. In the 17 patients with the obstruction pattern, conservative treatment was successful in one case and drainage treatment was successful in 10 of the remaining 16 cases. In cases with the delayed voiding pattern, conservative treatment was successful in six of seven cases and urinary drainage was successful in seven of eight cases. All three patients with the functional decline pattern underwent urinary drainage, which improved their renal function.

**TABLE 2:** Relationship between MAG_3_ pattern, treatment and outcomes.

Number of patients	MAG3 pattern	Number of patients	Treatment	Number of patients	Results	Number of patients
44	Non‐function	9	Non‐drainage	7	Nothing particular	7
					Failure	0
			Drainage	2	Improvement above 10% renal function	0
					Nothing improve	2
	Obstruction	17	Non‐drainage	1	Nothing particular	1
					Failure	0
			Drainage	16	Improvement above 10% renal function	10
					Nothing improve	6
	Delay	15	Non‐drainage	7	Nothing particular	6
					Failure	1
			Drainage	8	Improvement above 10% renal function	7
					Nothing improve	1
	Decline	3	Non‐drainage	0	Nothing particular	0
					Failure	0
			Drainage	3	Improvement above 10% renal function	3
					Nothing improve	0

Abbreviation: MAG_3_, [^99m^Tc]Tc‐mercaptoacetyltriglycine scintigraphy.

We also assessed the relationship between CT contrast enhancement patterns and treatment outcomes (Table 3). Twenty‐six patients underwent contrast‐enhanced CT, which revealed an asymmetric enhancement pattern in both kidneys in 18 cases. We provided conservative treatment in eight of these 18 patients and in four of the remaining eight patients with balanced contrast enhancement. We performed urinary drainage in 10 of the 18 patients with an asymmetric enhancement pattern and in four of the eight cases with balanced contrast enhancement. Within the group of 18 patients with the asymmetric enhancement pattern, conservative treatment was successful in eight patients, and renal function improved by more than 10% in seven of the remaining 10 patients. Conservative treatment was successful in all four cases with balanced contrast enhancement, and urinary drainage was successful in one of these cases.

**TABLE 3:** Relationship between contrast laterality, treatment and outcomes.

Number of patients	CE	Number of patients	Asymmetry	Number of patients	Treatment	Number of patients	Results	Number of patients
44	+	26	+	18	Non‐drainage	8	Nothing particular	8
							Failure	0
					Drainage	10	Improvement above 10% renal function	7
							Nothing improve	3
			−	8	Non‐drainage	4	Nothing particular	4
							Failure	0
					Drainage	4	Improvement above 10% renal function	1
							Nothing improve	3
	−	18						

Abbreviation: CE, contrast enhancement.

## 4 Discussion

In this study, we sought to determine if the MAG_3_ excretion pattern can identify patients with MUO who do not require urinary drainage. We found that 14 of 15 patients with MUO who were treated conservatively did not experience troublesome urinary events. The remaining patient developed new left hydronephrosis with lower back pain during follow‐up, so conservative treatment might not necessarily have been a failure in this patient.

Two studies have shown that findings on MAG_3_ scintigraphy can predict the efficacy of urinary drainage for hydronephrosis. Delayed tissue transit time on MAG_3_ scintigraphy predicts improvement of renal function after pyeloplasty in patients with ureteropelvic junction obstruction [10]. In a study of patients with asymptomatic ureteral stones, the grade of obstruction correlated with stone size but not with serum creatinine, grade of hydronephrosis or stone location [11], suggesting that the degree of hydronephrosis on ultrasonography is insufficient to assess obstruction. In the present quantitative review, contrast‐enhanced CT did not predict the success of urinary drainage for a symmetric pattern, with consideration that renal function did not improve after drainage in three of four patients. However, drainage improved renal function in seven of 10 patients with an asymmetric pattern in both kidneys (Table 3). These findings suggest that contrast‐enhanced CT is of limited value in terms of guiding the selection between urinary drainage and conservative treatment for patients with MUO.

The indications for urinary drainage in patients with MUO are controversial. The conservative treatment option is challenging in patients with hydronephrosis. While percutaneous nephrostomy offers effective urinary diversion in cases of MUO, MAG_3_ scintigraphy can serve as a complementary tool to assess functional necessity prior to intervention, thereby potentially reducing overtreatment [12]. However, one report recommends initial conservative care in infants with non‐obstructive hydronephrosis [13]. Ureteral stent placement often results in loss of quality of life for the patient [14]. Urinary drainage also imposes physical, emotional and financial burdens on patients. Therefore, preventing unnecessary urinary drainage benefits patients with MUO. Physicians who find hydronephrosis often ask urologists to perform urinary drainage. In a cervical cancer study, urinary drainage was performed in 145 of 179 patients with hydronephrosis [15]. The initial treatment for hydronephrosis was percutaneous nephrostomy in 77 patients and a ureteral stent in 68 patients. These treatments failed in 21.9% of patients. The overall complication rates of percutaneous nephrostomy (55.7%) and a ureteral stent (61.0%) were similar when adjusted for duration of treatment; the most common complication requiring intervention was urinary tract infection (29.2% vs. 27.3%) followed by haematuria (23.6% vs. 22.1%). Medical oncologists are more likely to recommend decompression for hydronephrosis in asymptomatic patients with a poor prognosis [16]. In our study, urinary drainage did not always improve renal function in patients with MUO, especially those who showed the non‐function pattern on MAG_3_ scintigraphy, suggesting that use of this imaging modality could avoid unnecessary urinary drainage.

The problem with MAG_3_ scintigraphy is that there needs to be more consistency in test performance and interpretation across centres and among diagnostic radiologists [17]. In our quantitative review, the MAG_3_ scintigraphy scans were obtained at a single centre and interpreted by the same experienced nuclear medicine physician.

When MAG_3_ scintigraphy revealed a non‐function excretion pattern, we achieved a 100% success rate with conservative treatment and no success with urinary drainage. This finding has encouraged us to opt for conservative treatment wherever possible. Our quantitative review also showed that the opportunity for conservative treatment may vary from case to case when the MAG_3_ excretion pattern is obstruction, delayed voiding, or functional decline. Notably, we found that renal function did not improve in six patients with an obstruction pattern despite urinary drainage, indicating a limitation of MAG_3_ scintigraphy. Therefore, shared decision‐making regarding urinary drainage or conservative treatment is essential while assessing the possibility of future surgery or chemotherapy, physical performance status and patient preference. For example, patients scheduled for chemotherapy may benefit from urinary drainage, and those scheduled for BSC may be more suitable for conservative treatment.

This case series has several important limitations. First, it was conducted as a retrospective observational review, which may have introduced selection bias and limited control over confounding factors. Second, the small number of cases—especially in the conservatively managed group—limits the ability to draw broadly applicable conclusions. Furthermore, as a descriptive case series without formal statistical analysis, this work does not offer strong evidence or establish causal relationships. The findings should be regarded as exploratory. To address these limitations, we are planning a prospective study to better define the clinical criteria for urinary drainage decision‐making using MAG_3_ scintigraphy.

In conclusion, based on the findings of this quantitative review, we recommend conservative treatment for patients with MUO if MAG_3_ scintigraphy shows a non‐functioning pattern.

## Ethics Statement

The study protocol was approved by the Clinical Research Ethics Committee of Gunma University Hospital (approval number 2114) and conducted in accordance with the Declaration of Helsinki.

## Conflicts of Interest

The authors declare no conflicts of interest.

## Data Availability

Data available on request from the authors.

## References

[1] A. M. Ganatra and K. R. Loughlin , “The Management of Malignant Ureteral Obstruction Treated With Ureteral Stents,” Journal of Urology 174 (2005): 2125–2128. 16280741 10.1097/01.ju.0000181807.56114.b7

[2] J. Asakawa , T. Iguchi , S. Tamada , et al., “Outcomes of Indwelling Metallic Stents for Malignant Extrinsic Ureteral Obstruction,” International Journal of Urology 25 (2018): 258–262. 29194771 10.1111/iju.13500

[3] J. Miyazaki , M. Onozawa , S. Takahashi , et al., “The Resonance Metallic Ureteral Stent in the Treatment of Malignant Ureteral Obstruction: A Prospective Observational Study,” BMC Urology 19 (2019): 137. 31881875 10.1186/s12894-019-0569-y PMC6935232

[4] J. Prentice , T. Amer , A. Tasleem , and O. Aboumarzouk , “Malignant Ureteric Obstruction Decompression: How Much Gain for How Much Pain? A Narrative Review,” Journal of the Royal Society of Medicine 111 (2018): 125–135. 29648512 10.1177/0141076818766725 PMC5900840

[5] K. Itoh , “99mTc‐MAG3: Review of Pharmacokinetics, Clinical Application to Renal Diseases and Quantification of Renal Function,” Annals of Nuclear Medicine 15 (2001): 179–190. 11545186 10.1007/BF02987829

[6] H. J. Lim and S. H. Choi , “Assessment of Individual Renal Function Using 99m Tc‐MAG3 Renography,” In Vivo 36 (2022): 206–211. 34972716 10.21873/invivo.12692 PMC8765135

[7] A. Faure , K. London , and G. H. Smith , “Early Mercaptoacetyltriglycine (MAG‐3) Diuretic Renography Results After Pyeloplasty,” BJU International 118 (2016): 790–796. 27105017 10.1111/bju.13512

[8] A. T. Taylor , D. C. Brandon , D. de Palma , et al., “SNMMI Procedure Standard/EANM Practice Guideline for Diuretic Renal Scintigraphy in Adults With Suspected Upper Urinary Tract Obstruction 1.0,” Seminars in Nuclear Medicine 48, no. 4 (2018): 377–390. 29852947 10.1053/j.semnuclmed.2018.02.010 PMC6020824

[9] N. Oriuchi , Y. Onishi , H. Kitamura , et al., “Noninvasive Measurement of Renal Function With 99mTc‐MAG3 Gamma‐Camera Renography Based on the One‐Compartment Model,” Clinical Nephrology 50 (1998): 289–294. 9840316

[10] S. H. Song , S. Park , S. Y. Chae , D. H. Moon , and K. S. Kim , “Predictors of Renal Functional Improvement After Pyeloplasty in Ureteropelvic Junction Obstruction: Clinical Value of Visually Assessed Renal Tissue Tracer Transit in,” Urology 108 (2017): 149–154. 28595935 10.1016/j.urology.2017.05.044

[11] F. Wimpissinger , C. Springer , A. Kurtaran , W. Stackl , and C. Türk , “Functional Aspects of Silent Ureteral Stones Investigated With MAG‐3 Renal Scintigraphy,” BMC Urology 14 (2014): 3. 24397735 10.1186/1471-2490-14-3 PMC3909333

[12] R. Dagli and P. Ramchandani , “Percutaneous Nephrostomy: Technical Aspects and Indications,” Radiologic Clinics of North America 49, no. 5 (2011): 111–127. 10.1055/s-0031-1296085 PMC3312169 23204641

[13] A. G. Hester , A. Krill , E. Shalaby‐Rana , and H. G. Rushton , “Initial Observational Management of Hydronephrosis in Infants With Reduced Differential Renal Function and Non‐Obstructive Drainage Parameters,” Journal of Pediatric Urology 18 (2022): 661.e1–e6. 10.1016/j.jpurol.2022.07.023 35989171

[14] H. B. Joshi , A. Stainthorpe , R. P. MacDonagh , F. X. Keeley , A. G. Timoney , and M. J. Barry , “Indwelling Ureteral Stents: Evaluation of Symptoms, Quality of Life and Utility,” Journal of Urology 169 (2003): 1065–1069, Discussion 9. 12576847 10.1097/01.ju.0000048980.33855.90

[15] H. E. Botkin , K. N. Faidley , B. T. Loeffler , S. L. Mott , E. K. Hill , and B. A. Erickson , “Longitudinal Outcomes From Conservative Management of Cervical Cancer Associated Ureteral Obstruction,” Urology 158 (2021): 208–214. 34582886 10.1016/j.urology.2021.09.007

[16] E. S. Hyams and O. Shah , “Malignant Extrinsic Ureteral Obstruction: A Survey of Urologists and Medical Oncologists Regarding Treatment Patterns and Preferences,” Urology 72 (2008): 51–56. 18372019 10.1016/j.urology.2008.01.046

[17] D. DiRenzo , A. Persico , M. DiNicola , S. Silvaroli , G. Martino , and P. LelliChiesa , “Conservative Management of Primary Non‐Refluxing Megaureter During the First Year of Life: A Longitudinal Observational Study,” Journal of Pediatric Urology 11 (2015): 226.e1–e6. 10.1016/j.jpurol.2015.05.007 26165191

